# Patients Affected by Unmethylated O(6)-Methylguanine-DNA Methyltransferase Glioblastoma Undergoing Radiochemotherapy May Benefit from Moderately Dose-Escalated Radiotherapy

**DOI:** 10.1155/2017/9461402

**Published:** 2017-10-12

**Authors:** Paolo Tini, Valerio Nardone, Pierpaolo Pastina, Giuseppe Battaglia, Clelia Miracco, Lucio Sebaste, Giovanni Rubino, Alfonso Cerase, Luigi Pirtoli

**Affiliations:** ^1^Sbarro Health Research Organization, Temple University, Philadelphia, PA, USA; ^2^Unit of Radiation Oncology, University Hospital of Siena, Siena, Italy; ^3^Department of Medicine, Surgery and Neurological Sciences, University of Siena, Siena, Italy; ^4^Unit of Pathological Anatomy, Department of Medicine, Surgery and Neurological Sciences, University of Siena, Siena, Italy; ^5^Unit of Neuroradiology, University Hospital of Siena, Siena, Italy

## Abstract

**Purpose:**

To compare the therapeutic results of two radiotherapy (RT) dose schedules in combined temozolomide- (TMZ-) RT treatment in newly diagnosed glioblastoma (GB), according to the O(6)-methylguanine-DNA methyltransferase (MGMT) methylation status.

**Material and Method:**

Patients received either standard (60 Gy) or moderately escalated dose (70 Gy) radiotherapy (RT) with concomitant and adjuvant TMZ between June 2006 and October 2013. We retrospectively evaluated the therapeutic effectiveness of RT schedules in terms of Overall Survival (OS) and Progression-Disease Free Survival (PDFS) analyzing the MGMT methylation status.

**Results:**

One hundred and seventeen patients were selected for the present analysis. Seventy-two out of the selected cases received the standard RT-TMZ course (SDRT-TMZ) whereas the remaining 45 underwent the escalated schedule (HDRT-TMZ). The analysis according to the MGMT promoter methylation status showed that, in unmethylated-MGMT GB patients, HDRT-TMZ and SDRT-TMZ groups had different median OS (*p* = 0,01) and PDFS (*p* = 0,007), that is, 8 months and 5 months for the SDRT-TMZ group and 14 months and 9 months for the HDRT-TMZ group, respectively. No difference in survival outcomes was found in methylated MGMT patients according to the two RT schedules (*p* = 0,12).

**Conclusions:**

In our experience, unmethylated-MGMT GB patients benefited from a moderately escalated dose of RT plus TMZ.

## 1. Introduction

Glioblastoma is the most frequent primary brain tumor (≥50% out of all the cases of primary tumors in the brain) with an incidence of about five new cases per 100,000 per year. Despite aggressive multimodal treatments, the prognosis of this disease remains poor with 5-year survival outcomes barely reaching 5%. Postsurgery RT plus TMZ chemotherapy is presently the backbone of the management of patients affected by GB [1].

A wide characterization of GB by multiple omics platforms has recently improved our knowledge of the molecular bases underlying GB aggressiveness [2, 3]. Nevertheless, in the clinical setting only the methylation status of the O(6)-methylguanine-DNA methyltransferase (MGMT) promoter, that is, a DNA repair enzyme that causes resistance to alkylating agents such as TMZ [4, 5], plays a practical role. The MGMT promoter methylation positive status has a highly significant predictive role of response to TMZ combined with RT [1, 5], whereas unmethylated MGM is considered an inherent prognostic indicator for patients with GB with a particularly poor survival [6, 7]. However, a more limited positive impact on survival results of the RT and TMZ combination was demonstrated also for these last patients [5]. More aggressive approaches may therefore be warranted for the latter category, even if those including very intensive TMZ administration failed to show a survival advantage [8]. We could not find any contribution particularly dealing with RT intensification. The present analysis is addressed to this topic, retrospectively comparing patients treated in our institution with a moderate RT dose escalation (70 Gy) plus TMZ [9] with those undergoing standard RT (60 Gy) plus TMZ, taking into account the MGMT methylation status.

## 2. Material and Method

We obtained ethics approval of the study and a signed informed consent by each patient for the anonymous use of clinical and treatment data. All the adopted procedures were in accordance with the ethical standards of the Helsinki Declaration (1964, amended most recently in 2008) of the World Medical Association.

### 2.1. Patient Series

We analyzed the medical records from our institutional brain tumor database containing 222 patients affected by GB (Grade IV–WHO Classification [10]), consecutively referred to the Radiation Oncology Unit for postoperative RT-TMZ, after the pathologic diagnosis (CM), from February 2007 to July 2014. The MGMT gene promoter methylation status was assessed using a methylation-specific Polymerase Chain Reaction (PCR), as previously reported [11]. Briefly, genomic DNA was extracted from paraffin-embedded tumor sections and treated with sodium bisulfite using the EZ DNA Methylation-Gold kit (HISS Diagnostics, GmbH, Freiburg, Germany). Primer sequences were used to detect methylated and unmethylated MGMT promoter sequences. PCR products were separated on 2% agarose gel. A glioma cell line with a completely methylated MGMT promoter and peripheral blood mononucleated cells served as positive and negative control samples, respectively. A methylation percentage of 5% was used as a cut-off value: samples with methylation < 5% and >5% were classified as unmethylated and methylated, respectively.

### 2.2. Extent of Surgical Resection

The extent of surgical resection was obtained by the description of surgical procedures and the postoperative CT-MR imaging (72 h and 30 days after surgery) and classified as follows: biopsy (B), subtotal resection (STR), and gross total resection (GTR).

### 2.3. Selection Criteria

**Figure 1:**
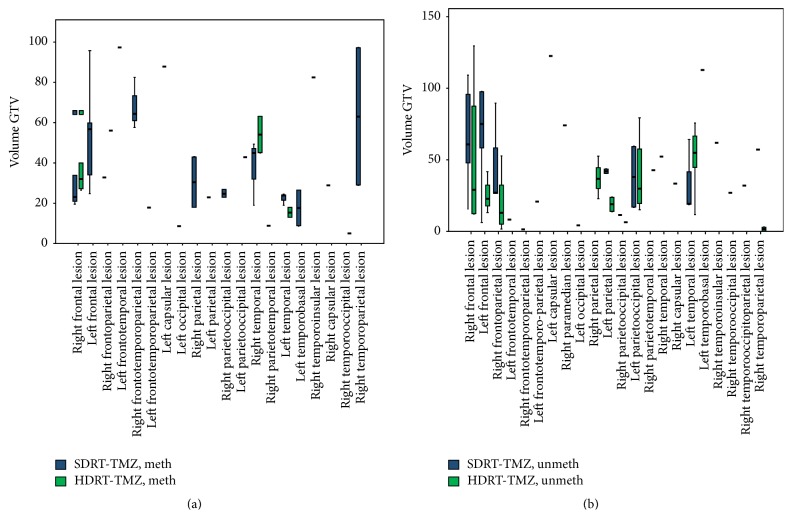
Anatomical distribution and GTV volume (expressed in cm^3^) of GB lesions treated with different RT dose scheme (chi-square test; *p* = 0,41) in methylated (a) and unmethylated (b) patients.

Only patients with unifocal GB have been considered for the present evaluation. All patients undergoing palliative whole brain irradiation for multifocal or very large GBs and with a Karnofsky Performance Status (KPS) score below 70% were excluded. Further, only patients whose Planning Target Volume (PTV, ≤115 ml) was compatible with the constraints we adopt for a boost up to 70 Gy, according to the previously published institutional protocol [9], were included in this study. Clinical and pathological data, including extent of surgery, prior to RT start, the baseline MRI (i.e., preresection or biopsy) study, were available in all cases. The MRI scan was obtained with a standard protocol, as follows: T1, T2, and FLAIR (Fluid Attenuated Inversion Recovery) acquisitions, DWI (Diffusion-Weighted Imaging) axial sequences (5 mm slices thickness/5.5 mm separation), and T1-gadolinium-enhanced scans, in axial, coronal, and sagittal planes. After the repetition of this exam before irradiation, all patients initiated the RT-TMZ adjuvant treatment within 4–8 weeks after surgery, according to the protocol defined by Stupp et al. [1]. The patients received either standard dose treatment* (SDRT: 59,4–60 Gy)* or moderate dose escalation* (HDRT: 69,4–70 Gy)* according to the selection criteria specified above. They were assigned to one or the other of these schedules based on the clinical judgement of the responsible radiation oncologists (PT, VN, PP, GB, LS, and GR) mainly taking into account the volume and the region of the lesion (Figures 1(a) and 1(b)) and adjacency to critical brain regions.

### 2.4. Radiotherapy Treatment Planning

*SDRT: 59,4–60 Gy.* The Clinical Target Volume (CTV) was contoured on CT and postoperative MRI image fusion and included residual tumor mass (T1 gadolinium-enhanced lesion) and/or postoperative cavity (i.e., GTV) plus a 15–20 mm margin without consideration for peritumoral edema. Volume contouring took into account anatomical barriers, such as ventricular spaces, cranial bones, and the midline except for the region of the corpus callosum. An isotropic margin of 5 mm was added around to obtain the Planning Target Volume (PTV-1). RT was delivered with a Linear Accelerator 6–10 MeV beam and 3D-Conformal or Intensity Modulated techniques up to a planned total dose of at least 59,4 Gy and with a standard fractionation (1,8–2 Gy/day for 5 days per week).

*HDRT: 69,4–70 Gy.* A dose boost up to 69,4–70 Gy was delivered to the selected cases, as defined above, according to the previously published institutional protocol, respecting OAR constraints in CNS [12]. Briefly, in patients without progression and relevant toxicity during the standard course of 59,4–60 Gy, a PTV-2 was created on GTV adding a margin of 5 mm; also, this boost was delivered with standard fractionation (2 Gy/day for 5 days per week).

*Chemotherapy.* All patients received also TMZ, concurrently administered* per os* during RT, according to Stupp's protocol (daily TMZ 75 mg/m^2^ during the RT course, for 6 weeks in SDRT and for 7 weeks in HDRT), followed by the sequential TMZ schedule (150–200 mg/m^2^ for 5 days every 28 days) until disease progression or complete response after 12 cycles.

### 2.5. Follow-Up

After the completion of RT and concurrent TMZ administration, patients entered a scheduled follow-up program. Brain MRI scans were repeated at 4 weeks, 12–16 weeks, and then every 6 months or in any case showing clinical signs suggesting progressive disease (PD). Taking into account the fact that no patient of this series received antiangiogenic treatment, PD after RT-TMZ treatment was assessed using the RANO Criteria [13]. A diagnosis of pseudoprogression was made in cases showing an increase in tumor size and/or T1-contrast enhancement within 3–6 months after the end of concomitant RT-TMZ, without worsening of neurological status and with stabilization or resolution in subsequent further MRIs studies. Imaging findings suggestive of radionecrosis were recorded. All the MRI examinations were revised for the compilation of this paper by a neuroradiologist (AC). General and neurological examinations and blood counts and chemistry were obtained every three months.

### 2.6. Analyzed Parameters, Survival End-Points, and Statistical Analysis

All the considered parameters were categorized as follows: patients' age at diagnosis (<50 ys and >50 ys), KPS (100–80 and = 70); extent of surgery (GTR: gross total resection; B-STR: biopsy or subtotal resection), MGMT status (methylated and unmethylated); RT dose (SDRT, 59,4–60 Gy, and HDRT, 69,4–70 Gy). In order to reduce bias selection due to the retrospective setting of analysis, we performed a cross-tab analysis (chi-square test) according to age, KPS, extent of surgery, tumor location, MGMT methylation status, and radiological response of SDRT-TMZ versus HDRT-TMZ patients groups.

We estimated PDFS and OS with the Kaplan-Meier method. The univariate survival analysis was used to identify the prognostic parameters. We used the log-rank test to assess the significance of survival differences for the considered parameters (*p* values ≤ 0,05 were considered as statistically significant). We also performed a multivariate analysis (Cox regression) to quantify the relationship between survival and potential predictors, in order to identify a subgroup of independent factors significantly related to survival. All the statistical analyses were performed with the SPSS 15.0 software package for Windows.

## 3. Results

Out of the 117 patients selected for this study, the MGMT promoter methylation status was, respectively, methylated (methMGMT) and unmethylated (unmethMGMT) in 48 (41%) and in 69 patients (59%).

The median OS of this whole series was 13 months, OS rate at 6 months and at 12 months being, respectively, 82,6% and 54,4%. Median PDFS was 9 months, with 6-month and 12-month rates of 62,6% and 38,7%, respectively.

Statistically significant prognostic factors for OS and PDFS at the univariate analysis were KPS, extent of surgical resection, and MGMT status (Table 1).

The multivariate analysis confirmed that KPS = 70 (HR: 2,424; 95% CI: 1,082–3,652; *p* = 0,001) and B-STR (HR: 1,783; 95% CI: 1,451–4,449; *p* = 0,001) and unmeth-MGMT status (HR: 3,088; 95% CI: 1,887–5,054; *p* = 0,001) were independently associated with a shorter OS and PDFS. Out of the whole series, in HDRT-TMZ and SDRT-TMZ groups, the median OS and PDFS times were similar: 12 months and 7 months for the SDRT-TMZ group and 14 months and 10 months for the HDRT-TMZ group.

**Figure 2:**
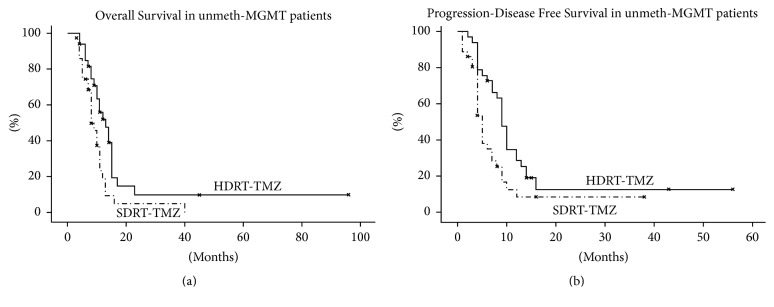
Overall Survival (a) and Progression-Disease Free Survival (b) (Kaplan-Meier method) according to standard (SDRT-TMZ) versus escalated (HDRT-TMZ) RT dose (log-rank test *p* value < 0,05) in unmethylated patients.

**Figure 3:**
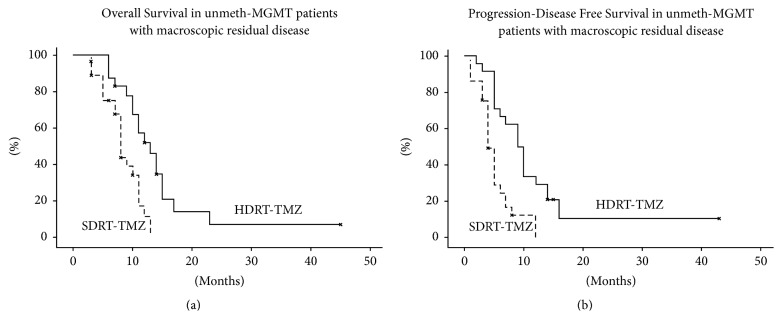
Overall Survival (a) and Progression-Disease Free Survival (b) (Kaplan-Meier method) according to standard (SDRT-TMZ) versus escalated (HDRT-TMZ) RT dose (log-rank test *p* value < 0,05) in unmethylated patients with incomplete resection.

A subgroup survival analysis for MGMT methylation status and extent of resection was performed. In unmeth-MGMT patients (48 pts), HDRT-TMZ and SDRT-TMZ groups had different median OS (*p* = 0,01) and PDFS (*p* = 0,007): 8 months and 5 months for the SDRT-TMZ group and 14 months and 9 months for the HDRT-TMZ group, respectively (Figure 2). No difference in OS and PDFS between HDRT-TMZ and SDRT-TMZ groups was found according to residual disease. Conversely, different OS (*p* = 0,001) and PDFS (*p* = 0,005) between HDRT-TMZ and SDRT-TMZ schemes, respectively, were found in unmeth-MGMT patients group with macroscopic residual disease (B/SRT) (Figure 3).

The RT dose was confirmed as an independent prognostic factor at multivariate analysis in unmeth-MGMT patients unadjusted (HR: 2,090; CI 95%: 1,151–3,795) and adjusted for extent of resection (HR 2,807; CI 95%: 1,186–4,567). No significant difference in distribution of the other prognostic factors was found between these two groups (Table 2). In terms of radiological response, the HDRT-TMZ group had a better response compared to the SDRT-TMZ one: an objective response (complete response + partial response) was demonstrated in 51,5% (14/33) and 13,9% (5/36) cases, respectively (*p* = 0,031, Table 2).

Differently, no difference in survival outcomes was found in meth-MGMT patients according to the different RT-TMZ schemes used (for OS: *p* = 0,12 and for PDFS: *p* = 0,23), even when analyzed by extent of surgical resection (data not reported).

## 4. Discussion

The treatment standard of GB includes maximal safe surgical resection followed by RT with concurrent and sequential TMZ (RT-TMZ) [1].

Response to treatment depends on the methylation status of the promoter of MGMT. In clinical practice, MGMT status is presently one of the most important biomarkers for prognostic stratification of GB patients, along with other molecular features, such as Isocitrate Dehydrogenase 1 (IDH1) mutational status, which are presently considered of the same clinical relevance [14, 15]. The MGMT promoter methylation status is associated not only with a different response (PDFS, OS) to RT-TMZ, but also with either treatment modality alone [7]. Meth-MGMT GB patients with a good performance status (ECOG 0-1) have a considerable response to RT-TMZ treatment. One study reports a median survival of 24.7 (ECOG 0)–16.4 (ECOG 1) months and a 2-year survival rate of 66–48,1%, whereas the corresponding results for unmeth-MGMT GBs are 12,9 (ECOG 0)–9,7 (ECOG 1) months and 2-year survival rates of 31% and 14,6%, respectively [16]. Considering that 90% of relapse occurs in the primary site, especially in unmeth-MGMT GB patients [17], this subgroup may theoretically benefit from increased RT dose schedules. The neurooncological community presently accepts a RT dose prescription of 60 Gy in RT-TMZ treatment of GB, delivered with conformal external beam techniques on partial-brain volumes, in five weekly fractions of 1,8–2,0 Gy [1]. However, mathematical modeling previsions consider the possibility of a substantially improved local control of GB with intensified dose-fractionation schedules [18]. In the clinical setting, this possibility has been widely investigated with unclear results. Tanaka et al. compared 60 and 61 patients with GBM who received 60 Gy and 80–90 Gy conformal RT, respectively, and suggested a survival benefit for patients treated with high dose [19], but most experiences failed to demonstrate a benefit for RT doses > 60 Gy delivered with external beam techniques [20, 21]. Also hypofractionation (often without TMZ chemotherapy) [22, 23] or other RT techniques, such as brachytherapy [24] or radiosurgery (SRS) [25], did not give appreciable results. A previous experience of our institution [9] with a protocol-driven RT-TMZ schedule and high-dose stereotactic radiation boost included 123 patients, 25 of whom received 70 Gy on a reduced volume, after a selection based on tumor size (≤5 cm major diameter). This series included also a minority of anaplastic astrocytomas; however, out of the 18 GBs that received 70 Gy boost, 2- and 3-year OS results were 39% and 22%. This treatment was well tolerated and no high-grade complication was detected during the follow-up, even if no systematic attempt was made for the diagnosis of asymptomatic brain RT damage. Based on this experience, assuming a potential and safe improvement in the treatment effectiveness by escalating the total RT dose without increasing the fraction size in selected patients, we continued to adopt this treatment protocol and analyzed the achieved results in the present study. Out of the overall selected series of 117 GB patients, the dose escalation (HDRT-TMZ) did not improve the survival outcomes. Retrospectively adopting a prognostic stratification according to the MGMT methylation status, we also found that PDFS and OS results of meth-MGMT patients were not influenced by the effect of HDRT-TMZ. In unmeth-MGMT patients, instead, the use of HDRT-TMZ schedule significantly improved both PDFS (*p* = 0,007) and OS (*p* = 0,01), compared to SDRT-TMZ. This finding did not seem to be related to a different distribution of the HD versus SDRT patients' characteristics in terms of age, extent of resection, and lesion localization (shown in Table 2(a)). However, the survival advantage seems to be related to a good treatment response, as shown by the radiological evaluation after treatment in HDRT-TMZ group (Table 2(a)). In these patients, the effect of HDRT in terms of survival gain is particularly significant when an extensive surgical resection was not accomplished. This is in contrast with the widely reported experience that the extent of residual disease after surgery is related to prognosis and patients with incomplete resections fared worse than those macroscopically resected [26, 27]. This generally agreed statement was the subject of a recent, very sophisticated analysis [28], showing that for any GB patient each prognostic covariate (i.e., age, KPS, extent of resection, and RT and TMZ treatment accomplishment) may have a predictive impact on survival. It could be expected, on these grounds, that the RT dose escalation up to 70 Gy (and perhaps the prolongation for a week of the concurrent TMZ-RT administration in unmeth-MGMT patients) may demonstrate a positive influence on local control of the disease and survival, especially when its influence is not covered up by the major effect of a macroscopically complete surgical resection. This possibility seems compatible with our results.

## 5. Limitations of the Study

The retrospective design, the adopted selection criteria, and the small number of patients are some pitfalls of the present analysis. IDH assessment was not reported because it was not performed across all patients. Lacking IDH assessment, even in presence of a small number of secondary glioblastomas, could reduce prognostic stratification of present analysis.

## 6. Conclusions

To the authors' knowledge, this is the first study reporting a possible benefit for unmeth-MGMT GB population from a RT moderate dose escalation with standard fractionation and the concurrent use of TMZ. These findings deserve further investigations and prospective trials may be devised on these bases.

## Figures and Tables

**Table 1:** Clinical (age = age at diagnosis, KPS = Karnofsky Performance Status), treatment (GTR = macroscopic gross total resection, B/STR = biopsy or subtotal tumor resection, dose RT = total dose for radiotherapy treatment, HDRT = 70 Gy, and SDRT = 59,4–60 Gy), and biological (MGTM) prognostic factors (Kaplan-Meier method, survival analysis).

		Number of patients	OS	*p* value	PDFS	*p* value
			Median (months)		Median (months)	
Age	>50	100	12	0,09	9	0,30
	<50	17	17		9	
KPS	100–80	106	14	0,001	9	0,018
	=70	11	7		4	
Extent of surgery	GTR	32	22	0,02	12	0,005
	B/STR	85	11		7	
MGMT status	Methylated	48	25	0,0001	15	0,001
	Unmethylated	69	11		7	
RT DOSE	HDRT	45	14	0,22	10	0,12
	SDRT	72	12		7	

**Table:** (a) HDRT-TMZ and SDRT-TMZ patient's characteristic in methylated group (*n* = 48)

		HDRT patients (*n* = 12)	SDRT patients (*n* = 36)	Chi-square significance
KPS	100–80	12	32	*p* = 0,67
	=70	0	4	

Extent of resection	GRT	6	10	*p* = 0,53
	B/SRT	6	26	

Age	>50 ys	11	27	*p* = 0,33
	<50 ys	1	9	

Radiological response	Complete response	6	9	*p* = 0,65
	Partial response	3	6	
	Stable disease	1	9	
	Progression disease	2	12	

Radionecrosis		1	1	*p* = 0,78

**Table:** (b) HDRT-TMZ and SDRT-TMZ patient's characteristic in unmethylated group (*n* = 69)

		HDRT patients (*n* = 33)	SDRT patients (*n* = 36)	Chi-square significance
KPS	100–80	30	32	*p* = 0,35
	=70	3	4	

Extent of resection	GRT	9	7	*p* = 0,56
	B/SRT	24	29	

Age	>50 ys	29	33	*p* = 0,46
	<50 ys	4	3	

Radiological response	Complete response	7	3	*p* = 0,031
	Partial response	7	2	
	Stable disease	7	6	
	Progression disease	12	25	

Radionecrosis		1	0	*p* = 0,8
